# Validation of the Chinese version of the Clinical Assessment Interview for Negative Symptoms (CAINS): a preliminary report

**DOI:** 10.3389/fpsyg.2015.00007

**Published:** 2015-02-02

**Authors:** Raymond C. K. Chan, Chuan Shi, Simon S. Y. Lui, Karen K. Y. Ho, Karen S. Y. Hung, Joanna W. S. Lam, Ya Wang, Eric F. C. Cheung, Xin Yu

**Affiliations:** ^1^Neuropsychology and Applied Cognitive Neuroscience Laboratory, Key Laboratory of Mental Health, Institute of Psychology, Chinese Academy of Sciences, Beijing, China; ^2^University of Chinese Academy of Sciences, Beijing, China; ^3^Peking University Sixth Hospital, Beijing, China; ^4^ Institute of Mental Health, Peking University, Beijing, China; ^5^Key Laboratory of Mental Health, Ministry of Health, Peking University, Beijing, China; ^6^Castle Peak Hospital, Tuen Mun, Hong Kong, China

**Keywords:** negative symptoms, schizophrenia, validation

## Abstract

The present study aimed to examine the psychometric properties of the Chinese version of the Clinical Assessment Interview for Negative Symptoms (CAINS). We recruited 68 patients with schizophrenia from the Chinese setting. The findings showed a generally consistent two-factor structure with the original version, namely “expression” and “motivation–pleasure.” There is a minor cultural variation in perceiving these items in the Chinese culture. However, the present study demonstrated that the Chinese version of the CAINS appears to be a valid and reliable clinical tool for the assessment of negative symptoms in the Chinese setting.

## INTRODUCTION

Negative symptoms have long been considered a core feature of schizophrenia and constitute significant unmet needs in the management of this disorder (Marder et al., 2013). Effective treatment for this domain of psychopathology is lacking (Kirkpatrick et al., 2006), and these symptoms represent a clinically important target for drug development. Anhedonia, an inability to experience pleasure, is a core negative symptom in schizophrenia that presents great challenge to available treatment and has significant bearings on prognosis (Lewis et al., 2006; Silver et al., 2009). Recent advance in affective neuroscience and cognitive neuroscience now suggests negative symptoms, and anhedonia in particular, may be conceptualized as a two-facet construct involving anticipatory and consummatory components (Knutson and Greer, 2008; Kringelbach and Berridge, 2009). Consummatory pleasure is the ability to experience momentary pleasure (i.e., the feeling of liking) when an individual is directly engaging in an enjoyable activity; whereas anticipatory pleasure is the ability to experience a motivated and goal-directed behavior (i.e., the feeling of wanting) for a future pleasant event (Klein, 1984; Berridge et al., 2009). Motivation is always accompanied by hedonic experience, especially anticipatory experience of pleasure (appetitive pleasure; Knutson and Greer, 2008; Kringelbach and Berridge, 2009). Conventional clinical ratings and interviews such as the Positive and Negative Syndrome Scale (PANSS; Kay et al., 1987) and the Scale for the Assessment of Negative Symptoms (SANS; Andreasen, 1982) do not take into consideration this two-facet construct in their content.

The Clinical Assessment Interview for Negative Symptoms (CAINS) was developed to capture the experience of pleasure during the rating period vs. expectation of pleasure in the future (Horan et al., 2011). The CAINS has incorporated the anticipatory and consummatory components of experiential pleasure in its formulation of the self-reported information obtained through face-to-face interviews. Moreover, it also highlights emotional expression based on the items or behaviors observed during the interview. Affective flattening and alogia are categorized to the “expression” factor, whereas asociality, avolition, and anhedonia are categorized to the “experiential” factor (Horan et al., 2011). Together, the CAINS is a 13-item interview tool comprising a nine-item motivation and pleasure subscale and a four-item expression subscale. Preliminary and subsequent large-scale clinical validation supports a two-factor structure for the CAINS, namely the “expression” and the “motivation/pleasure” factors (Kring et al., 2013). This two-factor structure has been found to be stable in a German sample (Engel et al., 2014). The CAINS represents an important and novel addition to the tools available for assessing negative symptoms in schizophrenia and other psychotic disorders both theoretically and clinically. Theoretically the CAINS assesses domains that map onto both the phenomenology of schizophrenia and constructs that have been informed by affective neuroscience in emotion, motivation, and affect processing. Clinically, it provides a broader coverage of negative symptoms than some of the conventionally used instruments, i.e., making a distinction between anticipation and experience in the social, recreational, work, and educational domains (Barch, 2013). This tool will serve as an important and valid tool for the assessment of negative symptoms in schizophrenia in different stages of the illness.

However, preliminary data are only available for Caucasian patients with established schizophrenia (Kring and Elis, 2013; Engel et al., 2014). Little is known about its psychometric properties and reliability in non-Caucasian populations such as the Chinese, and nothing is known about the prevalence of negative symptoms in patients with first-onset schizophrenia assessed by this new tool. There is substantial evidence suggesting that cultural variation exists between Western and Eastern people. For example, Chinese culture emphasizes the internal and external harmonious conditions of self and environment as opposed to the emphasis on individualism in Western culture (Chan et al., 2012). Chinese people tend to be more emotionally reserved and hence expressing lower frequency and intensity of emotional experience as compared to Western people (Bond and Hwang, 1986; Tsai et al., 2006a,b). In particular, minor cultural variation in reporting experiential pleasure in the Chinese context has been demonstrated in both healthy individuals and patients with schizophrenia (Chan et al., 2010a, 2012). Chinese people tend to report more details on contextual factor rather than the abstract factor as compared to Western counterparts (Chan et al., 2010a, 2012).

Therefore, the purpose of the present study was to examine the psychometric properties of the Chinese version of the CAINS in the Chinese setting. Given the theoretical framework of the CAINS and initial evidence of cultural stability in some non-English-speaking populations, we hypothesized that the two-factor structure of the original version, i.e., “expression” and “motivation/pleasure,” would be replicated in the Chinese context.

## MATERIALS AND METHODS

### PARTICIPANTS

The total sample included 68 patients (46% males) with schizophrenia. Their mean age was 32.41 years (SD = 12.41), mean years of education was 12.29 years (SD = 3.26), and their mean duration of illness was 56.29 months (SD = 80.73). Their mean scores in the PANSS were: Positive: 9.90 (SD = 3.55); Negative: 18.67 (SD = 6.92); General symptoms: 26.77 (SD = 6.84). Their diagnoses were made by their treating psychiatrists using the Structured Clinical Interview for DSM-IV (SCID; First et al., 1996). Exclusion criteria included: comorbid diagnosis of major depressive disorder, substance dependence in the past 6 months, substance abuse in the past month. IQ less than 70, and a history of head injury or neurological disorder. The study was approved by the ethics committees of the Institute of Psychology, Chinese Academy of Sciences, the Institute of Mental Health, Peking University, and the New Territories West Cluster of the Hong Kong Hospital Authority. All participants gave written informed consent. The study was conducted in conformity with the declaration of Helsinki (http://www.wma.net/en/30publications/10policies/b3/index.html).

### MEASURES

The CAINS is an interview-based assessment covering the “expression” factor (items included vocal prosody, gestures, facial, and speech) and the “motivation/pleasure” factor (items included recreation, social and vocational expected pleasure and motivation). It is a semi-structured interview following the instruction manual provided by the original authors. These items were assessed on the basis of patients’ reports of experienced motivation, interest, and emotion, as well as reports of actual engagement in relevant social, vocational, and recreational activities. Pleasure was assessed in terms of the past-week pleasure and the expected pleasure. All items were rated on a scale of 0–4, with higher scores reflecting greater impairment. The time period covered by the interview was the past 7 days except for expected pleasure, which covers the next 7 days. The whole interview lasted for about 30 min.

Before carrying out the translation, the agreement of the authors of the original instrument (Kring et al., 2013) was obtained. We then performed a forward–backward translation of the latest version of the CAINS (Kring et al., 2013) to Chinese (both Mandarin and Cantonese) according to the guidelines proposed by Beaton et al. (2000). The two dialects are exactly the same in terms of its meanings despite the different vocabulary and habitual expression. That means, the Mainland Mandarin is written in form of simplified Chinese characters, whereas the Hong Kong Cantonese is written in form of traditional Chinese characters. These two dialects are with different pronunciations and have different habitual expressions. The CAINS was first translated into Chinese and then back-translated to English by a research assistant, who had not seen the original version. This back-translated version was compared to the original, and a revised second translation was made. The divergence observed between the back-translation and the original English version was identified and discussed until a final version was ready for the study. Clinical raters with master-degree in clinical psychology or fellowship in psychiatry with extensive experience in conducting patient symptom interviews (CS, SSYL, KCYH, KSYH, JWSL) were trained in the CAINS interview. Training included review of the manual, and ratings of seven videotaped assessments provided by the authors of the original instrument. The targeted competency level was an Intraclass Correlation Coefficient of ≥0.90 between the last two ratings of the videotaped assessments and the gold standard ratings provided by the authors of the original instrument.

Validation measures were administered to all participants. These included the PANSS (Kay et al., 1987), the SANS (Andreasen, 1983), the Scale for the Assessment of Positive Symptoms (SAPS; Andreasen, 1984), the Social and Occupational Functioning Assessment Scale (SOFAS; Goldman et al., 1992) for the assessment of clinical symptoms and social functioning.

On the other hand, self-reported checklists were administered to participants to assess their emotional experience and expression. The Emotional Expressivity Scale (EES; Kring et al., 1994; Chan et al., 2010b) is a 17-item self-report checklist that captures individual differences in emotional expressivity. It is scored on a six-point Likert-type scale (1 = never true to 6 = always true), with a higher score corresponding to a higher ability to express one’s emotional status. The Chinese version was used for the present study and has been validated to have a two-factor structure of “emotional suppression” and “emotional expression” in Chinese samples (Chan et al., 2010b).

The Temporal Experience of Pleasure Scale (TEPS; Gard et al., 2006; Chan et al., 2012) is originally an 18-item self-report questionnaire that comprises items to capture anticipatory and consummatory pleasure. It is rated on a six-point Likert-type scale (1 = very false for me, 6 = very true for me). Higher score indicates a higher ability to experience pleasure. The Chinese version of the TEPS used in this study was modified with the deletion of two items and the addition of four items, resulting in a total of 20 items. In a large undergraduate sample from China (Chan et al., 2012), the 20-item TEPS yielded a four-factor structure: “abstract anticipatory pleasure,” “contextual anticipatory pleasure,” “abstract consummatory pleasure,” and “contextual consummatory pleasure.” The Chinese version of the TEPS has been shown to possess robust psychometric properties and good discriminative validity (Chan et al., 2010a, 2012).

### DATA ANALYSIS

Data were analyzed with SPSS 18.0. First, we conducted an exploratory factor analysis (principle component analysis) on the CAINS items, and the scree plot showed that a two-factor structure was suitable. Second, we examined the item-total correlation using bivariate correlations (Pearson correlation), internal consistency for factors and total scale using reliability analysis (Cronbach’s alpha). Third, external validation was conducted by correlating CAINS factor scores with SANS total score, SAPS total score, PANSS positive score, PANSS negative score, PANSS general psychopathology score, TEPS total score, two factor scores, and four subscale scores, EES factor scores, and SOFAS total score using bivariate correlations (Pearson correlation).

## RESULTS

Exploratory factor analysis showed that KMO = 0.866, χ^2^ = 529.06, *p* < 0.001, which meant that the data were suitable for factor analysis. We extracted two factors, which explained 54.63% of the total variance. The two factors were named “expression” and “motivation and pleasure.” Two items [CAINS 1 (social, family relationships) and CAINS 5 (vocational, motivation)] which belong to the “motivation and pleasure” factor in the original version went to the “expression” factor in the present study (see Table 1 for factor loading). The factor scores were generated by adding corresponding item scores. Higher score corresponded to more serious symptoms.

**Table 1:** Factor structure and loading of the CAINS.

	**Expression**	**Motivation and pleasure**
CAINS12	**0.879**	0.191
CAINS11	**0.844**	0.332
CAINS13	**0.821**	0.307
CAINS10	**0.791**	0.362
CAINS1	**0.618**	0.289
CAINS5	**0.449**	0.267
CAINS4	0.268	**0.724**
CAINS3	0.303	**0.695**
CAINS7	0.263	**0.62**
CAINS6	0.196	**0.6**
CAINS8	0.205	**0.598**
CAINS9	0.207	**0.586**
CAINS2	0.381	**0.504**

The mean score of each item, each factor and the total score are presented in Table 2. The item-total score correlation is also presented for each item, which ranged from 0.58 to 0.81. The internal consistency (Cronbach’s alpha) for the “motivation and pleasure” factor, the “expression” factor and the whole scale were 0.85, 0.90, and 0.91 respectively.

**Table 2:** Descriptive statistics and reliabilities of the CAINS.

	**Mean**	**SD**	**Item-total correlation**	***α***
**Motivation and Pleasure**	13.91	6.30		0.85
CAINS2	1.91	1.07	0.69	
CAINS3	1.96	1.34	0.73	
CAINS4	2.19	1.35	0.73	
CAINS6	2.38	1.36	0.61	
CAINS7	1.49	1.13	0.66	
CAINS8	2.04	1.05	0.62	
CAINS9	1.97	1.36	0.62	
				
**Expression**	9.12	5.23		0.90
CAINS1	1.38	1.03	0.68	
CAINS5	1.91	1.21	0.58	
CAINS10	1.58	1.01	0.80	
CAINS11	1.35	1.01	0.81	
CAINS12	1.67	1.09	0.73	
CAINS13	1.23	1.00	0.77	
				
**CAINS**	23.03	10.39		0.91

Correlation analyses were conducted to examine the convergent and discriminate validity of the Chinese version of the CAINS (Table 3). Both the “motivation and pleasure” factor and the “expression” factor were significantly and positively correlated with the SANS total score, the negative symptoms subscale score and general psychopathology subscale score of the PANSS. These results suggested good convergent validity of the CAINS. Moreover, the “motivation and pleasure” factor also correlated with the “emotional suppression” subscale score of the EES. Both factors were negatively correlated with social function as measured by the SOFAS, suggesting good convergent validity. On the other hand, both the derived factors showed no significant correlation with the SAPS total score or the positive symptoms subscale score of the PANSS, indicating good discriminate validity.

**Table 3:** Convergent and discriminate validity of the CAINS.

	**Motivation and pleasure**	**Expression**
SANS	0.652^**^	0.887^**^
SAPS	0.334	0.246
PANSS Positive Symptoms	0.274	0.191
PANSS Negative Symptoms	0.622^**^	0.861^**^
PANSS General psychopathology	0.466^**^	0.527^**^
TEPS	–0.2	–0.159
Consummatory factor	–0.223	–0.229
Abstract consummatory	–0.011	–0.167
Contextual consummatory	0.103	0.064
		
Anticipatory factor	–0.123	–0.043
Abstract anticipatory	–0.181	–0.257
Contextual anticipatory	0.114	0.088
EES suppression factor	0.405^*^	0.292
EES expression factor	–0.16	–0.192
SOFAS	–0.554^**^	–0.715^**^

SANS, Scale for the Assessment of Negative Symptoms; SAPS, Scale for the Assessment of Positive Symptoms; PANSS, Positive and Negative Syndrome Scale; TEPS, Temporal Experience of Pleasure Scale; EES, Emotional Expressivity Scale; SOFAS, the Social and Occupational Functioning Assessment Scale ^*^p < 0.05; ^**^p < 0.01.

## DISCUSSION

The Chinese version of the CAINS showed a consistent two-factor structure, namely the “expression” and the “motivation–pleasure” factors, with the original version (Kring et al., 2013) and the German version (Engel et al., 2014). The internal consistency of the two subscales and the total scale was excellent, ranging from 0.85 to 0.91. The item-total correlations also ranged from 0.50 to 0.79, suggesting a very stable internal consistency and item-total correlation.

The significant correlation of the CAINS with the negative symptoms subscale score of the PANSS but not the positive symptoms subscale score demonstrated a strong convergent validity of the Chinese version. We only found both the “expression” and “motivation–pleasure” factors to be correlated significantly with the consummatory subscale but not the anticipatory subscale of the TEPS in our sample. However, our findings are consistent with the German version of the CAINS. Engel et al. (2014) also did not find any significant correlation of the CAINS with the anticipatory subscale score of the TEPS. We posited that this non-significant correlation might be due to the fact that the TEPS was designed primarily to capture physical pleasure as reflected by the items embedded in the scale (Gard et al., 2006). However, the CAINS covers a wide range of behavior touching on interpersonal, occupational, and social relationship rather than the physical aspect of pleasure, and hence, may have a wider variation for the observed association between the anticipatory subscale score of the TEPS. Other scales that better characterizes social and interpersonal deficits (Gooding and Pflum, 2014a,b) should be used to further examine this speculation in the near future. It is also noteworthy that the “motivation–pleasure” factor was significantly correlated with the “emotional suppression” factor of the EES. This suggests that patients with deficits in the “expression” and “motivation–pleasure” factors of the CAINS also showed poorer emotional expressivity. These findings support the convergent validity of the Chinese version of the CAINS.

However, it should be noted that items 1 (social, family relationships) and 5 (vocational, motivation) were loaded onto the expression factor with a factor loading of 0.62 and 0.45, respectively. These items were loaded onto the “motivation–pleasure” factor in its original and German versions (Kring et al., 2013; Engel et al., 2014). The loadings of these two items in the present sample were comparable to those reported in the original version (item 1: 0.33; item 5: 0.68) and German version (item 1: 0.43; item 5: 0.53), suggesting the possibility of cultural variations in perceiving these items in the Chinese culture. Traditional Chinese are group-oriented and tend to conform to social norms compared to their western counterparts who are more individual-oriented (Bond and Hwang, 1986). Confucianism has been the official state ideology throughout Chinese history and the model man in traditional Chinese society is the “gentleman-scholar” who anticipates trouble in advance and take steps to prevent it (Koo, 1989). Furthermore, nourishment of life has always been emphasized by the Chinese, including concern for job, health and the subsequent activities directed to enhancing qualify of life and health status (Koo, 1989). Family is particularly important for Chinese people who have distinguished from others in a hierarchical but interconnected relationship (Bond and Hwang, 1986). Maintaining a stable family relationship may be a proper way to express their emotional status. Similarly, showing motivation to search for a job or sustain a stable vocational status is also a way of expressing emotion and intention to maintain the family’s financial situation. However, since the present findings are preliminary, further research is needed to cross-validate our findings in a larger sample.

On the other hand, despite the fact that Chinese people are more emotionally reserved and expressing lower frequency and intensity of emotional experience as compared to Western people (Tsai et al., 2006a,b), the findings on the two factors loading on “expression” seem more or less similar to the original version of the CAINS. This finding suggests the “expression” factor is relatively stable in the current Chinese sample. Once again, given the preliminary nature of the current sample, further research is needed to cross-validate our findings in a larger sample.

The present study has several limitations. First, this preliminary study recruited a relatively small sample and made use of exploratory factor analysis, but the sample size is comparable to previous pilot studies done in the original and the German version. Second, the present study only used a limited set of self-reported checklists capturing the physical domain of anhedonia. A wide range of measures should be included to cover social and interpersonal deficits in further study to clarify the association of the CAINS and different domains of anhedonia in patients with schizophrenia. Finally, it should be noted that the CAINS is a relatively long interview that needs to be conducted by a trained psychiatrist or related professionals as compared to self-reported checklists, which may constrain its applicability in clinical settings.

Notwithstanding these limitations, this pilot study demonstrates robust psychometric properties of the Chinese version of the CAINS and suggests that this version is suitable for clinical and research purposes for the assessment of negative symptoms in patients with schizophrenia in the Chinese setting.

## AUTHOR CONTRIBUTIONS

Raymond C. K. Chan generated the idea, designed the study, interpreted the data and wrote up the first draft of the manuscript. Chuan Shi, Simon S. Y. Lui, Karen K. Y. Ho, Karen S. Y. Hung, Joanna W. S. Lam, collected the data and administered the CAINS, and commented the first draft of the manuscript. Ya Wang analyzed the data and helped write up the manuscript. Eric F. C. Cheung and Xin Yu commented and contributed significantly to the writing up of the first draft of the manuscript. All authors contribute to and have approved the final text.

### Conflict of Interest Statement

The authors declare that the research was conducted in the absence of any commercial or financial relationships that could be construed as a potential conflict of interest.
